# 胸腔镜肺段切除术对肺功能影响的研究进展

**DOI:** 10.3779/j.issn.1009-3419.2019.08.10

**Published:** 2019-08-20

**Authors:** 少龙 巨, 禹舜 高

**Affiliations:** 100021 北京，国家癌症中心，国家肿瘤临床医学研究中心，中国医学科学院北京协和医学院肿瘤医院胸外科 Department of Thoracic Surgery, National Cancer Center/National Clinical Research Center for Cancer/Cancer Hospital, Chinese Academy of Medical Sciences and Peking Union Medical College, Beijing 100021, China

**Keywords:** 肺肿瘤, 肺段切除术, 肺功能, 电视胸腔镜, Lung neoplasms, Segmentectomy, Pulmonary function, Video-assisted thoracoscopy

## Abstract

2018年美国国立综合癌症网络关于非小细胞肺癌（non-small cell lung cancer, NSCLC）指南指出，对于早期NSCLC，解剖性肺叶切除为首选方案。随着电视胸腔镜技术的发展，以胸腔镜为代表的胸外科微创手术在临床得到了广泛应用。胸腔镜肺段切除术已经成为早期NSCLC的治疗方案之一。临床研究发现相较于肺叶切除，亚肺叶切除在早期NSCLC治疗中也可取得相似的结果并保留更多的肺功能，但肺段切除术后患者肺功能的改变尚存争议。本文将重点对胸腔镜肺段切除术后患者肺功能改变的研究进展做一综述。

## Abstract (en)

The National Comprehensive Cancer Network (NCCN) clinical practice guideline of non-small cell lung cancer (NSCLC) in 2018 points out that anatomic pulmonary resection is a preferred option for early stage NSCLC. With the development of video-assisted thoracoscopy, minimally invasive thoracic surgery represented by thoracoscopy has been widely used in clinical practice. Video-assisted thoracoscopic segmentectomy has become one of the treatment options for early stage NSCLC. Clinical studies have found that sublobar resection can achieve similar results and preserve more pulmonary function in the treatment of early stage NSCLC compared with lobectomy, but the changes of pulmonary function after segmentectomy are still controversial. This article focuses on the research progress of pulmonary function changes in NSCLC patients after video-assisted thoracoscopic segmentectomy.

全球范围内肺癌仍然是所有癌症中发病率及死亡率最高的肿瘤，2018年据估计世界上新发肺癌210万例，死于肺癌的患者共180万例^[1]^。随着影像学技术发展，胸部计算机断层扫描（computed tomography, CT）检查可以更早的发现肺部病变，尤其是肺部磨玻璃结节，这导致了外科医生开始考虑能否通过亚肺叶切除实现肿瘤的完全切除。近年来，已经有相关研究^[2, 3]^表明，与标准的肺叶切除相比，亚肺叶切除可使患者获得相似的预后，同时有大量研究表明其在患者术后肺功能保留及恢复方面的优势。随着胸腔镜手术（video-assisted thoracoscopic surgery, VATS）技术的发展，通过胸腔镜微创方法进行非小细胞肺癌（non-small cell lung cancer, NSCLC）切除越来越受欢迎。通过胸腔镜行肺叶及肺段切除术的比例也大大增加，从2007年的11.6%到2015年其应用率已达60.6%。本文将从电视胸腔镜肺段切除的手术适应证、胸腔镜肺段切除术对肺功能的影响、肺段切除术后肺功能代偿及展望四个方面对目前研究进展做一综述。

## 1 胸腔镜肺段切除术的适应证

1995年肺癌研究小组（Lung Cancer Study Group, LCSG）的一项前瞻性多中心随机队列研究纳入肺叶及亚肺叶切除的247例T1-2N0期NSCLC患者，该研究比较了两组患者局部或远处复发率、5年生存率、围术期死亡率及术后肺功能差异，研究表明与肺叶切除相比，亚性肺叶切除可导致肺癌患者存在较高的术后复发率及死亡率^[4]^。从此以后NSCLC患者肺叶切除及肺门纵隔淋巴结清扫成为标准术式。

肺段切除根据肺组织气血双重供应的解剖学特点，在符合肿瘤学原则的前提下，以保留肺功能为目的，肺实质切除范围局限于一个或多个肺段。随着影像学技术的发展，周围型肺癌及磨玻璃结节检出率较以往明显增加。2018年底，美国国立综合癌症网络（National Comprehensive Cancer Network, NCCN）指南提出了解剖性肺段切除的手术指征，主要包括：①肺功能较差或有其他主要合并症不适于行肺叶切除者；②周围型肺癌（肿物位于肺外周1/3）患者至少满足下列条件之一者：a.原位腺癌；b.CT显示肺结节中磨玻璃成分≥50%；c.CT检测肿瘤倍增时间≥400 d^[5]^。手术相对禁忌证包括：严重肺气肿、间质性肺炎、广泛的胸腔黏连等。

胸腔镜肺段切除尤其适用于病灶小，不可直视或不可触及的可切除病变。尽管周围型肺癌可以通过术前CT定位后楔形切除实现肿瘤切除^[6-8]^。但是此方法不适用于深在的或者肿瘤不易于被CT定位的病灶（如肿瘤位于肺尖、肩胛骨前、靠近主要血管、贴近纵隔或横膈膜等）。另外CT引导定位可能导致气胸、出血、气栓、标记物脱落或移动、手术切缘不够等问题^[9, 10]^。相反，肺段切除可满足上述条件，可实现深在肿瘤的切除，保证足够的切缘^[11]^。

## 2 肺段切除对肺功能的影响

理论上来说，胸腔镜肺段切除术应该兼有胸腔镜手术微创的优点及肺段切除对肺功能影响小的优点。一项包含16项研究的系统综述认为：肺段切除确实较肺叶切除保留了更多的肺功能。在术后最初2个月及术后12个月，肺段切除组第1秒用力呼气容积（forced expiratory volume in one second, FEV_1_）较术前减少9%-24%及3%-13%，其减少量均较肺叶切除组少^[12]^。近期日本一项临床研究纳入了108例肺段切除术及208例肺叶切除术患者。研究者通过性别、吸烟情况、肺功能等因素进行匹配后两组分别入组了103例患者，比较了两组患者在不同术式后肺功能的改变情况。该研究表明：肺段切除组整体肺功能有更明显的保留（*P* < 0.001），肺段切组保留切除侧肺功能（48±21）%；肺段切除组同侧未手术肺组织肺功能显著增加（*P*=0.003），但肺叶切除组无明显增加（*P*=0.97），同时两组对侧肺功能均有代偿性增加（*P* < 0.001）^[13]^。另有一项病理分期为T1a期并纳入37例肺段切除患者及33例肺叶切除患者的回顾性研究表明，只有在术后最初2个月中肺段切除组肺功能恢复明显优于肺叶切除组，之后两组肺功能恢复未见明显差异。术后2个月用力呼气量（forced vital capacity, FVC）及FEV_1_恢复率在肺段及肺叶切除组分别为81.8% *vs* 70.7%（*P* < 0.001）、81.4% *vs* 71.0%（*P* < 0.01）。在术后7个月-12个月FVC及FEV_1_恢复率在肺段及肺叶切除组分别为93.4% *vs* 93.2%（*P*=0.97）、87.8% *vs* 88.8%（*P*=0.82）。术后超过12个月，所有的肺功能指标在两组之间均无显著差异^[14]^。但是Charloux等^[12]^认为，即使术后超过12个月，肺段切除组肺功能受影响程度较肺叶切除组仍然更轻，但是这种差异较小，对于需要多次手术的患者更有益。目前有大量研究^[15-17]^表明，肺段切除在保留肺功能方面有明显优势。

由于成年人肺间质不可再生，因此术后肺功能理论上主要取决于术中切除肺组织的多少^[18]^。在肺段切除患者中，Nomori等^[13]^比较了切除 < 2个肺段及≥2个肺段对患者肺功能的影响，结果显示随着肺段切除数量的增加，患者肺功能受影响程度逐渐增大并且肺功能恢复更慢。并且Macke等^[19]^也发现FEV_1_在肺段切除较多组（3段-5段）较肺段切除较少组（1段-2段组）下降更多（0.3 L *vs* 0.1 L, *P*=0.003），一氧化碳弥散量（diffusing capacity of the lung for carbon monoxide, DLCO）也存在相似的改变（2.4 mL/min/mmHg *vs* 1.3 mL/min/mmHg, *P*=0.015）。以上研究结果表明肺段切数量越多，对肺功能的影响程度越大。

然而有部分研究表明，胸腔镜肺段切除术在保留肺功能方面并不优于胸腔镜肺叶切除术。Deng等^[20]^一项纳入212例肺段切除术及2, 336例肺叶切除术的回顾性研究显示，两组患者术后肺功能无显著差异。这可能是因为该研究中实际进行术后肺功能差异比较时只对17例肺段切除术和51例肺叶切除术患者肺功能进行比较，样本含量较小可能导致统计结果无显著差异。Hwang等^[21]^的一项倾向性匹配研究比较了肺段切除组及肺叶切除组患者术后肺功能的改变，两组分别入组94例患者，其术后FEV_1_下降率无明显组间差异[肺段组（8.9±10.8）*vs* 肺叶组（11.0±13.1），*P*=0.36]。Suzuki等^[14]^的研究结论相似，他们认为两组术后长期而言肺功能无显著性差异的结果可能是因为肺叶切除术后肺组织代偿性增长的原因。

## 3 肺段切除术后肺功能代偿

2012年Butler等^[22]^报道称，1例女性患者行右全肺切除术后15年长期随访观察发现该患者肺功能逐渐恢复（FEV_1_较术预测值增加51%，FVC增加35%）。Nomori等^[13]^研究了103例肺段切除术患者及103例肺叶切除术患者术后肺功能的变化，研究发现肺段切除术后同侧剩余肺组织可代偿性增长（*P*=0.003），但是肺叶切除组同侧剩余肺组织未见明显代偿性增长（*P*=0.97），同时两组对侧肺组织均有显著性代偿性增长（*P* < 0.001）。其原因可能在于：第一，在肺叶切除组术前已经发生了肺组织代偿性增长，因此术后无进一步增长，因为肺叶切除组肿瘤体积较肺段切除组更大^[23]^；第二，剩余肺组织代偿增长可逐渐填补已切除肺组织的空间，这可能导致剩余肺叶支气管角度改变、气道扭转及气道阻力增加，这些因素均可影响肺通气及换气功能^[23, 24]^；第三，与肺段切除比较，肺叶切除术组对侧肺组织过度增长在一定程度上限制了同侧剩余肺组织的增长可能性^[23]^。

另有相关研究^[22, 25]^也表明同侧剩余肺组织和/或对侧肺组织的增长在一定程度上起到了代偿作用。此外，除外肺组织代偿性增长外，肺部血管在术后也有相似改变，使得通气血流比例相应提高，以上因素在NSCLC患者肺段切除术后肺功能恢复中扮演重要角色。需要注意的是，术中切割闭合器的使用可防止术后肺漏气，但也可能会限制术后剩余肺段的代偿性增长^[26, 27]^，因此外科医生在使用切割闭合器时应考虑到这一问题。

## 4 展望

大量研究表明，胸腔镜肺段切除在早期NSCLC患者术后肺功能保护中较胸腔镜肺叶切除更具优势。但是很少有学者研究术后早期，尤其是术后几天至几周内胸腔镜肺段切除术对患者肺功能的影响。其原因在于术后早期肺功能复查不是常规项目，患者依从性也会因为术后早期疼痛而降低，但是术后早期是并发症发生的主要时间段。另外尚需研究，是否胸腔镜肺段切除对于基础肺功能较差的患者更有益，这是否意味着扩大了肺功能方面的手术适应症。尽管目前胸腔镜肺段切除术受到欢迎，但是其长期预后及肿瘤相关预后仍具有争议。由于磨玻璃结节就实性成分比例不同，其肿瘤异质性也有所增加，胸腔镜肺段切除术的指征也更难以界定。胸腔镜肺段切除术难点在术中确定清扫淋巴结站数、对肺段相关解剖结构的认知、段间静脉的保护、手术切缘及段间平面的确定^[28]^。这些难点也限制了该术式的广泛普及和推广，在实践中进行经验总结将有利于上述问题的解决。

机器人辅助胸腔镜手术（robotic-assisted thoracoscopic surgery, RATS）已经开始进入我们的视野。尽管其普及程度仍然较低，但是关于机器人辅助胸腔镜肺段切除术的手术技术及手术效果方面的相关报道正在逐渐增多^[29, 30]^。与胸腔镜肺段切除术相似，其近期及远期肿瘤学结果及治疗费用等方面仍有待于进一步评估。目前大量的研究主要比较了胸腔镜肺段切除及胸腔镜肺叶切除之间的优劣，将来讨论热点将逐渐转移为胸腔镜肺段切除术及机器人辅助胸腔镜肺段切除术的比较^[31]^。
